# The Effect of Molecular Hydrogen on Functional States of Erythrocytes in Rats with Simulated Chronic Heart Failure

**DOI:** 10.3390/life13020418

**Published:** 2023-02-02

**Authors:** Anna Vyacheslavovna Deryugina, Darya Andreevna Danilova, Vladimir Viktorovich Pichugin, Yurii Dmitrievich Brichkin

**Affiliations:** 1 Department of Physiology and Anatomy, Institute of Biology and Biomedicine National Research, Lobachevsky State University of Nizhny Novgorod Address: 23 Prospekt Gagarina (Gagarin Avenue), 603950 Nizhny Novgorod, Russia; 2 Clinical Cardiac Surgery Hospital, Nizhny Novgorod Address: 209, Vaneeva Street, 603081 Nizhny Novgorod, Russia

**Keywords:** red blood cell (RBC), molecular hydrogen (H_2_), microcirculation, aggregation, malondialdehyde (MDA), catalase activity, electrophoretic mobility, lipid peroxidation

## Abstract

Molecular hydrogen has an anti-inflammatory and cardioprotective effect, which is associated with its antioxidant properties. Erythrocytes are subjected to oxidative stress in pathologies of the cardiovascular system, which is the cause of a violation of the gas transport function of blood and microcirculation. Therefore, our aim was to investigate the effects of H_2_ inhalation on the functional states of red blood cells (RBCs) in chronic heart failure (CHF) in rats. The markers of lipid peroxidation, antioxidant capacity, electrophoretic mobility of erythrocytes (EPM), aggregation, levels of adenosine triphosphate (ATP) and 2,3-diphosphoglyceric acid (2,3-DPG), hematological parameters were estimated in RBCs. An increase in EPM and a decrease in the level of aggregation were observed in groups with multiple and single H_2_ application. The orientation of lipoperoxidation processes in erythrocytes was combined with the dynamics of changes in oxidative processes in blood plasma, it was observed with both single and multiple exposures, although the severity of the changes was greater with multiple H_2_ inhalations. Probably, the antioxidant effects of molecular hydrogen mediate its metabolic action. Based on these data, we conclude the use of H_2_ improves microcirculation and oxygen transport function of blood and can be effective in the treatment of CHF.

## 1. Introduction

Chronic heart failure (CHF) is a complex syndrome that occurs as a result of structural and functional disorders affecting the ability of the heart to supply oxygen to tissue. Heart failure is the leading cause of morbidity and mortality and causes high health-related costs [1].

Despite the achievements of modern medicine, the prevalence and hospitalization of patients with this pathology increases annually. This is due to the current improvement of the treatment of other cardiovascular diseases (such as myocardial infarction), population aging and comorbidities and additional chronic diseases [2]. Therefore, the search for diagnostic criteria and therapeutic approaches is highly justified [3].

Hypoxia, accompanied by increased oxygen consumption, is a trait common to the development of chronic heart failure syndrome caused by various etiologies. Pathophysiological mechanisms of heart failure development include inflammation, myocardial injury, fibrosis, oxidative stress, hypertrophy and neurohormonal activation, which cause hemorheological disorders and inadequate blood flow in CHF [4].

Red blood cells (RBCs) provide the delivery of oxygen to tissues to meet metabolic needs [5] and are directly involved in the important process of vital activity—transcapillary metabolism [6].

Ischemic injuries resulting from hemodynamic changes provoke oxidative stress and, as a consequence, a change in the mechanical properties of erythrocytes, a decrease in their deformation, an increase in adhesion to the vascular wall. The result of these processes is hypoxia of tissue [7]. Over the last decades, multiple approaches have been identified for the therapeutic exploitation of medical gases [8] and one of them is molecular hydrogen (H_2_). H_2_ has antioxidant, anti-inflammatory and anti-apoptotic properties [9,10]. It has been shown that inhaling H_2_ caused an improvement in ischemia/reperfusion injuries of the brain [11] and in myocardial infarction [12].

Numerous publications of preclinical and clinical studies have demonstrated the beneficial effects of H_2_ in various diseases associated with increased oxidative stress. The cardioprotective effects of molecular hydrogen in patients were confirmed, which was associated with H_2_ antioxidant properties [10]. Oxidative stress may play a key role in the pathology of CHF, e.g., in https://openheart.bmj.com/content/5/2/e000814 (accessed on 15 December 2007) [13]. However, the mechanisms based on the action of molecular hydrogen on erythrocytes in the correction of CHF have not been studied.

Based on these previous studies, the objectives of this study were to investigate effects of H_2_ inhalation on the functional states of RBCs in chronic heart failure in rats.

## 2. Materials and Methods

### 2.1. Animal Model and Care

The research was approved by the Local Ethics Committee for conducting scientific research involving animals as research objects of the Lobachevsky State University on 9 October 2020, and conducted following the European Community guidelines (EEC Directive of 1986; 86/609/EEC). Male Wistar rats (*n* = 30) weighing 260 ± 20 g were obtained from SPF-vivarium of Laboratory Animals Genetic collections Center (LAGCC) (Nizhny Novgorod, Russia). Rats were acclimated for one week in plastic cages. Animals were raised under standard laboratory conditions (12 h light and dark cycle, 23 ± 3 °C temperature, and 50–60% humidity), food and water without restrictions. The study was performed in accordance with guidelines for animal research (ARRIVE guidelines 2.0) [14].

### 2.2. Verification of CHF Rats Model

CHF was induced as previously described [15] by intraperitoneal injection of 1% adrenaline (Epinephrine hydrochloride, Federal State Unitary Enterprise “Moscow Endocrine Plant”, Moscow, Russian Federation) at 0.3 mg/kg three times every 48 h [16]. The consequences of chronic epinephrine exposure include biventricular heart failure and ventricular remodeling, with clinical hyperadrenergic conditions, chronic heart failure develops. Significant cardiopulmonary disorders with the development of pulmonary edema occur as a result of the use of high doses of adrenaline [16]. Therefore, the use of adrenaline concentration is associated with the recommended dose causing CHF [17]. There were hypodynamia, languid, breathlessness, excessive salivation, red eyes, developed after the 2nd administration of drugs in this model, and an increase in the severity of clinical manifestations was noted after the 3rd administration of drugs. The cardiac function of rats was evaluated by echocardiographic measurement using an ultrasound Doppler system for animals (S12-4, Philips CX50, Holland) after anesthesia. The images were recorded in M-mode the day before the first injection of adrenaline and the day after the third injection of adrenaline to confirm the development of CHF in rats. Specialists collected and described the data in a blind manner. The data obtained confirmed the change in the structure and function of the heart: left ventricular end-diastolic dimension (LVEDD) (before the experiment = 5.957 ± 0.314 mm, after the third injection = 7.643 ± 0.141 mm), left ventricular end-systolic dimension (LVESD) (up to = 3.923 ± 0.274 mm, after the third injection = 5.096 ± 0.324 mm), right atrium vertical diameter (RAVD) (before the experiment = 3.618 ± 0.473 mm, after the third injection = 4.846 ± 0.227 mm), right atrium transverse diameter (RATD) (before the experiment = 5.171 ± 0.176 mm, after the third injection = 4.755 ± 0.139 mm), right ventricle (RV) (before the experiment = 2.182 ± 0.265, after the third injection = 2.673 ± 0.095 mm), left atrium (LA) (before the experiment = 4.267 ± 0.311, after the third injection = 4.949 ± 0.135 mm), interventricular septum (IVS) (before the experiment = 0.876 ± 0.238, after the third injection = 1.298 ± 0.164 mm), left ventricular posterior wall (LVPW) (before the experiment = 1.073 ± 0.145 mm, after the third injection = 1.495 ± 0.212 mm), RV thickness (before the experiment = 1.817 ± 0.199 mm, after the third injection = 2.517 ± 0.183 mm), mitral E/A peak velocity (E/A) (before the experiment = 5.112 ± 0.174, after the third injection = 3.786 ± 0.249), ejection fraction (EF) (before the experiment = 0.612 ± 0.033%, after the third injection = 0.497 ± 0.027%) and fractional shortening (FS) (before the experiment = 0.362 ± 0.025%, after the third injection = 0.215 ± 0.087%).

Histological studies of the rat’s heart indicated the presence of pathognomonic changes for CHF. Along with normal cardiomyocytes, there were destructive and hypertrophied forms, the nuclei were wrinkled, deformed, reduced in size, hyperchromic, displaced to the cell periphery, a plethora of vessels, pronounced infiltration and signs of fibrosis were also observed. Histomorphometry was estimated by the μvizo 103 transmitted light micro-imager (“LOMO”) at magnifications of 20× and 40×.

### 2.3. Experiment Grouping and Process

The day after the CHF simulation, the experimental animals were randomly divided into three groups with ten rats (*n* = 10) in each group. In the 1st research group, the animals were placed in ventilated boxes (24L) connected to the hydrogen gas generator (Bozon H_2_/O_3_, Odessa, Ukraine) and admitted to spontaneous breathing (2% H_2_, 96% air containing 21% O_2_) for 40 min a day for 5 days in a row. The portable gas analyzer of explosive and toxic gases and vapors “Hydrogen (H_2_)” (“Signal-4” Moscow, Russia) was installed in the box to control the concentration of gases. In the 2nd research group, rats breathed a gas–hydrogen mixture (2% H_2_) for 40 min once (the day after the CHF simulation) then air was supplied to the box for the next 4 days. In the control group, rats were placed in the box and fresh air was ventilated continuously for the same duration as the hydrogen inhalations exposure time.

### 2.4. Blood Collection

Blood for the study was obtained from the sublingual vein after the CHF induction, on the 1st, 3rd, 7th and 14th day after it, according to the standard procedure.

### 2.5. Materials

#### 2.5.1. The Electrophoretic Mobility of Erythrocytes (EPM)

As previously described, we measured the EPMs of rats RBCs by calculating the velocity of microscopic particles in a 10 mM tris-HCl phosphate-buffered saline of pH 7.4 under the influence of an external electric field (12 mA) [18]. To determine the value of this indicator, we used the formula in our modification:

U = S × g × χ/T × 0.08,(1)

where U—EPM, S—100 μm, g—0.04 cm (cross section of the camera), χ—specific electric conductivity, T—the time of cell movement, sec. 

#### 2.5.2. Erythrocyte Aggregation

An effective method to assess microcirculation disorders is to assess the aggregation of erythrocytes under microscopy of dilute blood in the Goryaev’s camera (LLC Minimed, Bryansk, Russian Federation) [19]. The degree of aggregation severity was assessed by the result obtained by the ratio of the number of non-aggregated erythrocytes (a 1:10 blue dextran solution was used to activate aggregation) to the total number of erythrocytes (%).

Aggregation index (%) = 100 − (the number of non-aggregated RBCs/total RBCs × 100),(2)

#### 2.5.3. Adenosine Triphosphate (ATP)

The main energy parameter is the concentration of ATP in red blood cells. The evaluation of this indicator in the suspension of washed erythrocytes was carried out by the presence of inorganic phosphorus (Pi) in hydrolyzed erythrocytes on a photometer photoelectric KFK-3 -“ZOMZ” (JSC ZOMZ, Sergiev Posad, Russian Federation) at a wavelength of 660 nm using the non-enzymatic method described by us earlier [20].

#### 2.5.4. 2,3-Diphosphoglyceric Acid (2,3-DPG)

Glycolysis of erythrocytes differs from other cells in the production of a significant amount of 2,3-DPG, which is a heterotropic allosteric modulator of hemoglobin binding to oxygen and, as a consequence, a regulator of tissue gas exchange. The concentration of 2,3-DPH was also determined by the non-enzymatic method by the increase in inorganic phosphate in the supernatant of hemolyzed erythrocytes (Pi1) and after ashing (Pi2) according to the calibration curve, in a standard solution of KH_2_PO_4_.

The concentration of 2,3-DPG was measured using the following equation:

[2,3-DPG] = (100 × Pi1 − 10 × Pi2)/2,(3)

#### 2.5.5. Lipid Peroxidation

Colored trimetin complex with maximum absorption at a green light filter was used to determine the concentration of MDA expressed in nmol/mL of erythrocytes [21]. The results were estimated as the following:

[MDA] = D × 50/1.56 (4)

where D—optical density, 50—dilution, 1.56—the molar extinction coefficient MDA. 

The lipid peroxidation products concentration was determined by the absorption of a monochromatic light flux in the ultraviolet region of the spectrum by a lipid extract. The amount of diene conjugates (DC), triene conjugates (TC) and Schiff bases (SB) are extracted in heptane-isopropanol fractions. Measurement of optical densities (E) was performed on a SF-2000 spectrophotometer (CJSC OKB Spectrum, St. Petersburg, Russian Federation). DC, TC and SB concentration was calculated from the relative values of E232\E220, E278\E220, E400\E220 and in relative units.

#### 2.5.6. Antioxidant Capacity

The antioxidant capacity was determined spectrophotometrically by measuring the catalase activity using the method of Beers and Sizer [22] and expressed as the quantity of μmol of H_2_O_2_ converted by the enzyme per unit time (min) in mg of hemoglobin (Hb)—μmol/gHb × min. Catalase activity was calculated using an extinction coefficient of the test sample immediately (E_1_) and 20 s after adding H_2_O_2_ (E_2_):

Catalase activity = (lg E_1_/E_2_ × 120,000)/Hb (5)

#### 2.5.7. Hematological Parameters

The study of hematological parameters was carried out on the hematological analyzer “Abacus Junior” 30ND (Diatron, Austria), the number of red blood cells (RBCs), the hemoglobin concentration (Hb) and the average volume of erythrocytes (MCV) were determined.

### 2.6. Statistical Analysis

Data are presented as arithmetic mean values and standard deviations. The distribution was checked for compliance with the normal law by calculating the Kolmogorov–Smirnov criterion. It was revealed that for all the studied indicators, the type of distribution of the data obtained corresponds to normal, and therefore the subsequent analysis for determining statistically significant differences was carried out using the Student’s *t*-test. Statistical analysis was estimated using the BIOSTAT (Analyst-Soft Inc., Walnut, CA, USA) and Microsoft Excel for Windows (MS Office 2016 (16.0.5266.1000), MSO (16.0.5266.1000), Version 64, Santa Rosa, CA, USA) application software packages using one-dimensional statistics methods.

## 3. Results

### 3.1. The Electrophoretic Mobility of the Cell

**Figure 1:**
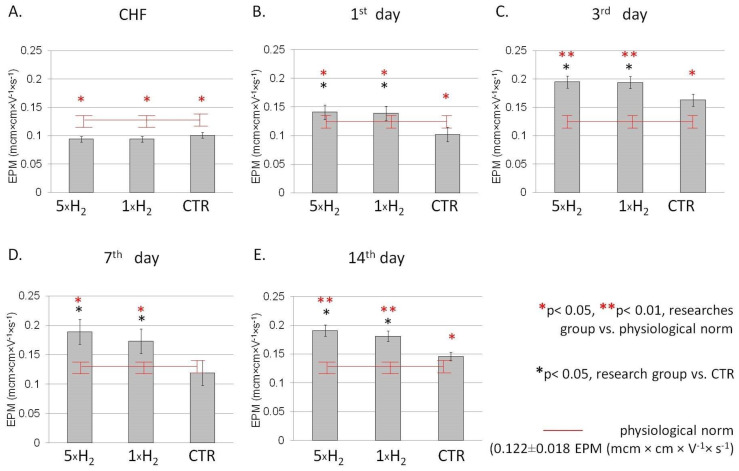
Dynamics of the EPM (mcm × cm × V^−1^ × s^−1^) in the blood of rats with simulated CHF against the background of the action of molecular hydrogen. Note: EPM dynamics with multiple (5 × H_2_), single (1 × H_2_) H_2_ inhalation and in the control (CTR) group after the simulation of CHF (**A**), on the first (**B**), third (**C**), seventh (**D**) and 14th (**E**) days after the simulation of CHF. Data are presented as mean values ± SD. * *p* ≤ 0.05 vs. CTR group (Student’s *t*-test); * *p* ≤ 0.05 vs. physiological norm (Student’s *t*-test) EPM: Electrophoretic mobility; CHF: chronic heart failure.

The physicochemical state of the cell membrane along with the composition of the environment surrounding the cell has a decisive influence on the electrokinetic properties of the cell. An indicator of the electrokinetic properties of the cell is the electrophoretic mobility of the cell and the value of the ζ –potential. The experimentally measured value is EPM, which is recognized as an approximate measure of its net surface charge density of the membrane. In our study, EPM significantly decreased in rats with simulated CHF, compared to the indicators of intact animals (Figure 1). Inhalation of H_2_ caused an increase in EPM relative to the control group at all stages of the study. At the same time, on the first and third days, single and repeated exposure to H_2_ determined the same type of action and an increase in EPM by 29–34% of the values of the control group. On the 7th and 14th day of the study, the increase in EPM was more pronounced with repeated exposure and amounted to 58–34% comparatively to the control group, respectively, whereas with a single exposure, the severity of the changes was less, although it remained higher than the control group (Figure 1). 

### 3.2. The Aggregation

**Figure 2:**
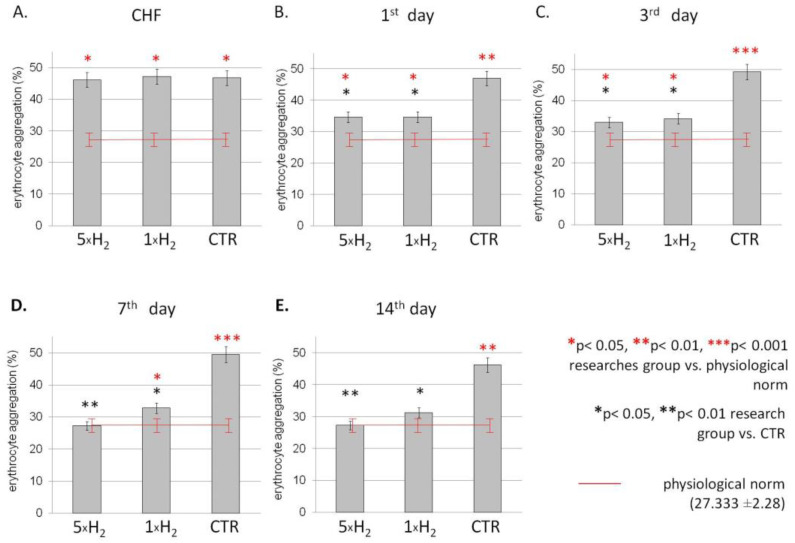
Dynamics of the erythrocyte aggregation (%) in the blood of rats with simulated CHF against the background of the action of molecular hydrogen. Note: Dynamics of the rat’s erythrocyte aggregation (%) with multiple (5 × H_2_), single (1 × H_2_) H_2_ inhalation and in the control (CTR) group after the simulation of CHF (**A**), on the first (**B**), third (**C**), seventh (**D**) and 14th (**E**) days after the simulation of CHF. Data are presented as mean values ± SD. * *p* ≤ 0.05 vs. CTR group (Student’s *t*-test); * *p* ≤ 0.05 vs. physiological norm (Student’s *t*-test); CHF—chronic heart failure.

The study of the aggregation properties of erythrocytes showed an increase in the level of aggregation in the group with simulated CHF comparatively to the intact group (Figure 2). Further, using molecular hydrogen inhalation, the aggregation of erythrocytes decreased throughout the experiment relative to the control group, in which aggregation on the contrary increased. We demonstrated, using the H_2_, a significant decrease in aggregation indicators from the values of the control group by 26% on the first day; following this, aggregation continued to decrease and by the third day it decreased by 33 and 31% with multiple and single exposure to H_2_, respectively. By the seventh day, there was a difference between groups with single and multiple use of H_2_, a predominance of a lower level of aggregation in the group with repeated exposure to H_2_. On the 14th day, aggregation in the group with multiple exposures to H_2_ was restored to the values of the intact group; with a single exposure, aggregation also decreased, while in the control group this indicator exceeded the values of the intact group by 69% (Figure 2).

### 3.3. The Energetic Metabolism

Two of well-studied markers of energetic metabolism are ATP and 2,3-DPG. We found reduced levels of ATP in erythrocytes of rats with simulated CHF compared to healthy animals (Table 1). The concentration of ATP was elevated by the first day after inhalation of H_2_ in both research groups. Further, as a result of multiple exposures to H_2_, the concentration of ATP in erythrocytes had increased by 3, 7 and 14 days and was statistically higher than the values of the control group. At the same time, in the group with a single use of H_2_, the growth of this indicator comparatively to the control was recorded on the 14th day, with a decrease to the control values on the 3rd–7th day. By day 14, the ATP level in both research groups was restored to the values of the physiological normal, while in the control group it remained lowered.

Concentration of 2,3-DPG decreased on the 3rd and 14th day of registration in the control group and in the group with a single H_2_ exposure, relative to the physiological norm. 2,3-DPG of erythrocytes regulates gas exchange, lowering the affinity of hemoglobin to oxygen. The elevated 2,3-DPG of rats with multiple exposures to H_2_ relative to the control group on the 3rd and 14th day and exceeding values of the healthy group on the 14th day testify a decrease in the affinity of hemoglobin to oxygen in erythrocytes by a right shift of the oxygen equilibrium curve, enhancing oxygen delivery to tissues (Table 1). 

### 3.4. Oxidative Stress Markers

Oxidative stress markers often correlate with the activity of the enzyme link of the antioxidant system. We studied the dynamics of changes in the concentration of malondialdehyde (MDA) as a marker of oxidative stress and catalase activity which serves as one of the main enzymes of the antioxidant system. MDA and catalase are present in erythrocytes in sufficiently high concentrations that can be easily measured in most laboratories, providing valuable information about the oxidative stress. MDA concentration decreased in both H_2_ exposure groups relative to the values of the control group by the 1st day and remained reduced with multiple exposures to H_2_ with a significant decrease in the indicator on the 14th day of registration (Table 2). Catalase activity tended to increase in both experimental groups, but significantly increased with repeated use of H_2_ relative to the control values on days 3 and 14 (Table 2).

The orientation of the processes of lipoperoxidation in erythrocytes was combined with the dynamics of changes in oxidative processes in blood plasma. Our study indicates the reduction in Schiff bases (SB) from the third day of research in groups receiving molecular hydrogen therapy. At the same time, the dynamics coincided with both single and multiple exposures to H_2_, although the severity of the changes was greater in the first research group (Table 3).

### 3.5. Hematological Parameters

To test the effect of molecular hydrogen on hematological parameters, we analyzed the quantitative and qualitative composition of red blood. The study of the red blood cell count (RBCs), hemoglobin (Hb) and the mean corpuscular volume (MCV) showed that the use of H_2_ led to a decrease in these indicators on the first day in both groups with H_2_ inhalations (Table 4). On the 3rd and 14th days, a decrease in indicators was recorded with multiple exposures to H_2_ relative to the control group, which amounted to a drop in RBC by 16% and 19%, respectively, and a drop in Hb by 12% and 25%, respectively. MCV was also reduced with repeated inhalations of hydrogen by the 14th day by 7% relative to the values of the control group, in which this indicator remained elevated throughout the experiment.

## 4. Discussion

In this study, we compared how different duration of inhalations of molecular hydrogen affects changes in the functional state of erythrocytes. Decline of red blood cell parameters was observed in the group of rats with simulated CHF: erythrocyte aggregation increased, and the mean corpuscular volume increased. These changes can lead to circulatory disorders, impaired blood oxygenation in capillaries, decreased oxygen transport function of blood and oxygen starvation of tissues increased. Aggregation of erythrocytes prevents the entry of cells into narrow capillaries and promotes their shunting through wider vessels, bypassing capillary networks [23], which in turn significantly affects metabolic processes at the capillary level and contributes to the development of tissue hypoxia. At the same time, a compensatory increase in the volume of RBCs has been shown in response to a decrease in oxygenation. However, the oxygen release slows down in such cases [24]. In addition, the process of erythrocyte maturation and hemoglobin synthesis is disrupted in chronic heart failure, the level of some pro-inflammatory cytokines increases, namely tumor necrosis factor-alpha (TNF-α), which inhibits normal hematopoiesis, and the reception and synthesis of erythropoietin (EPO) is also disrupted [25]. In conditions of CHF, it is possible that a decrease in the volume fraction of shaped elements can be considered as a compensatory reaction aimed at optimizing blood circulation and improving the rheological properties of blood. Inhalations containing 2% molecular hydrogen caused a more significant decrease in the number of red blood count to the control, a decrease in the amount of Hb and MCV of red blood cells, which is likely to increase the efficiency of circulation in the circulatory system.

In return, the surface charge of erythrocytes plays an essential role in the mechanism of aggregation of erythrocytes. [26]. Decrease in the surface charge of erythrocytes causes the formation of erythrocyte aggregates [27]. The improvement of circulation associated with a decrease in erythrocyte aggregation may be due to the increase in the erythrocyte membrane electronegativity detected by us in the study under the action of H_2_. It is possible that under the action of H_2_, the structure of erythrocyte membranes stabilizes due to a decrease in lipoperoxidation processes. In our experimental model, this fact is reflected in a decrease in the concentration of MDA in red blood cells. Application of H_2_ by inhalation contributes to reduce oxidative stress and reduced myocardial damage [28]. The action of hydrogen as an antioxidant, due to its ability to diffuse rapidly through membranes, selectively reduces the levels of cytotoxic ROS formed during oxidative stress, neutralizing reactive oxygen species—hydroxyl radical OH– and peroxynitrite ONOO– [29]. Moreover, H_2_ does not affect other ROS which play an important role in maintaining cell REDOX balance and cell function [8,11]. In addition, molecular hydrogen can reduce oxidative stress through its action on the antioxidant system, stimulating the growth of components of the antioxidant system, including hemoxygenase-1 (HO-1), superoxide dismutase, activity of catalase and myeloperoxidas (MPO) [30]. It is possible that the antioxidant effects of molecular hydrogen mediate its metabolic action. It has been shown that with a decrease in cell acidosis, glycolysis processes increase [31]. The use of H_2_ in our study led to an increase in the ATP concentration in erythrocytes. 

The ATP formed during glycolysis serves primarily as a substrate of Na(+)/K(+)—ATPase and Ca(2+)—ATPase, which support the membrane potential of erythrocytes to preserve the integrity of the membrane and the biconcave shape of RBCs, ensuring normal functioning and oxygen transport function. It has been shown that an increase in calcium concentration enhances aggregation, changes intermolecular interactions, transforms the shape of erythrocytes due to the inclusion of the calcium signaling pathway and dephosphorylation of key proteins of the membrane cytoskeleton [32,33,34]. Therefore, an increase in the concentration of ATP could contribute through the operation of the Ca (2+)—ATPase to the restoration of normal calcium levels in red blood cells and a decrease in their aggregation. In addition, an increase in ATP content in erythrocytes leads to phosphorylation of spectrin, ankyrin and band 4.1 proteins, weakening protein–protein interactions and affecting cell plasticity in general [35]. 2,3-DPG reversibly interacts with cytoskeleton proteins [36]. These processes contribute to an increase in the deformation of erythrocytes.

Apparently, under the application of H_2_, processes develop in erythrocytes that optimize their rheological properties, which is necessary for adequate micro- and macrocirculation of blood. It should be noted that the deformation of erythrocytes can promote the release of both NO [37] and ATP from cells [38], which can stimulate the production of NO in blood vessels [39]. Nitric oxide released by erythrocytes determines vasodilatation of resistive microvessels [40,41]. In the hypoxia zone, erythrocytes are released and are able to activate purinergic receptors on the vascular endothelium, as a result of which the secretion of nitric oxide and other factors results in the dilation of blood vessels [5]. Through an increase in NO, erythrocytes participate in the regulation of local vascular resistance and thereby ensure the effectiveness of microcirculation. The importance of such an influence in the conditions of CHF increases even more, since the possibilities of regulating the volume component of the microcirculation are limited. Thus, we can talk not only about the antioxidant effect of H_2_, but also about its metabolic and antihypoxic effects.

Discussing the mechanisms of action of H_2_, it is necessary to consider not only its direct, but also its indirect effect, since the effects of H_2_ in our study manifest themselves on the 14th day, a considerable time after the H_2_ inhalation. It has been shown that molecular hydrogen specifically neutralizes not only the hydroxyl radical (•OH), which has a high cytotoxic effect, but also peroxynitrite (ONOO−) [9,42]. A variety of protein factories controlling transcription are nitrolated (-O-NO_2_) or nitrosolated (-S-NO_2_). Thus, the reduction of these factors can control gene expression [43]. In addition, the results of our previous studies indicate that the change in EPM allows us to characterize the development of stress reactions and the inclusion of adaptive processes of the organism. The registered primary decrease in EPM is associated with an increase in the level of circulating catecholamines in the blood and an increase in the sensitivity of adrenoreceptors to them [44]. During the deployment of the stress reaction, catecholamines, activating the release of adrenocorticotropic hormone (ACTH), stimulate an increase in the level of adrenal cortex hormones in the blood, which leads to an increase in cortisol levels and an increase in EPM, i.e., we observe the second phase of the stress reaction, which is accompanied by recovery processes and increased resistance of the body [45]. 

The results of the experiments suggest that the stress reaction progresses with the development of CHF, which manifests itself in a decrease in EPM. The degree of changes in EPM in simulated CHF might correlate with the involvement of stress-implementing systems of the body. Molecular hydrogen seems to be able to increase the body’s resistance by limiting the stress reaction.

Considering activation of the sympathoadrenal system (SAS) [46,47] and hypersympathicotony makes a significant contribution to the pathogenesis of CHF and has a significant impact on the course and prognosis of the disease [48,49]; limiting the stress reaction with molecular hydrogen is an important finding of the study.

Thus, in our study, we demonstrate that the effect of inhaling H_2_ leads to an increase in EPM, ATP concentration, 2,3-DPG and a decrease in the level of aggregation and lipoperoxidation, which is probably explained both by the antioxidant effect of H_2_ on the processes occurring in erythrocytes, and by the indirect action of molecular hydrogen through the restriction of the stress reaction, as evidenced by the change in EPM.

## 5. Conclusions

In conclusion, according to the results obtained, it can be said that the use of H_2_ has a beneficial effect on the surface charge, metabolism and aggregation of red blood cells, which improves microcirculation and oxygen transport function of blood and can be effective in the treatment of CHF. Furthermore, our study could be useful in developing new therapeutic methods that include using molecular hydrogen in cardiovascular diseases. Further research should focus on evaluating the effect of molecular hydrogen depending on the duration of use and the number of inhalations.

## Figures and Tables

**Table 1:** Concentration of ATP and 2,3-DPG in erythrocytes of rats with simulated CHF.

Indicator	Group	Day of the Experiment			
		1	3	7	14
ATP (μmol Pi/mL)	5 × H_2_	1.120 ± 0.063	2.724 ± 0.357 *	3.961 ± 0.090 *	2.166 ± 0.298 *
	1 × H_2_	1.944 ± 0.210 *	1.464 ± 0.119	3.814 ± 0.146	2.480 ± 0.756 *
	CTR	0.960 ± 0.240	1.380 ± 0.162	3.500 ± 0.187	1.534 ± 0.124
2,3-DPG (μmol Pi/mL)	5 × H_2_	13.403 ± 1.587	13.261 ± 1.444 *	10.404 ± 1.060	16.914 ± 1.460 *
	1 × H_2_	12.940 ± 0.601	5.428 ± 1.489	10.090 ± 0.180	10.510 ± 1.970
	CTR	11.595 ± 0.816	8.278 ± 2.885	11.532 ± 2.815	9.343 ± 0.614

Note: ATP: adenosine triphosphate; 2,3-DPG: 2,3-diphosphoglyceric acid. 5 × H_2_—animals received multiple H_2_ exposures; 1 × H_2_—animals received single H_2_ exposure; CTR—control group. Data are presented as mean values ± SD. * *p* < 0.05, vs. control group (Student’s *t*-test). Indicators of healthy animals (physiological norm): concentration of ATP—2.131 ± 0.085 (μmol Pi/mL), concentration of 2,3-DPG—14.234 ± 1.013 (μmol Pi/mL).

**Table 2:** Concentration of MDA and catalase activity in erythrocytes of rats with simulated CHF.

Indicator	Group	Day of the Experiment			
		1	3	7	14
MDA (nmol/mL)	5 × H_2_	0.539 ± 0.270 *	2.378 ± 0.147	2.077 ± 0.107	0.549 ± 0.131 *
	1 × H_2_	0.404 ± 0.110 *	2.692 ± 0.530	2.570 ± 0.189	1.263 ± 0.153
	CTR	2.563 ± 0.270	2.863 ± 0.354	2.269 ± 0.172	1.539 ± 0.485
Catalase activity (units/gHb×min)	5 × H_2_	0.492 ± 0.105	0.954 ± 0.166 *	0.691 ± 0.183	1.542 ± 0.210 *
	1 × H_2_	0.803 ± 0.194	0.786 ± 0.071	1.040 ± 0.295	1.269 ± 0.227
	CTR	0.547 ± 0.128	0.618 ± 0.140	0.617 ± 0.271	0.729 ± 0.327

Note: MDA: malondialdehyde. 5 × H_2_—animals received multiple H_2_ exposures; 1 × H_2_—animals received single H_2_ exposure; CTR—control group 5 × H_2_—animals received multiple H_2_ exposures; 1 × H_2_—animals received single H_2_ exposure; CTR—control group. Data are presented as mean values ± SD. * *p* < 0.05, vs. control group (Student’s *t*-test). Indicators of healthy animals (physiological norm): concentration of MDA—0.513 ± 0.071 (nmol/mL), the level of catalase activity—1.14 ± 0.143 (units/gHb × min).

**Table 3:** The levels of diene, triene conjugates and Schiff’s bases in the blood plasma of rats with simulated CHF.

Indicator	Group	Day of the Experiment			
		1	3	7	14
DC (relative unit)	5 × H_2_	0.195 ± 0.008	0.206 ± 0.006	0.156 ± 0.009	0.210 ± 0.021
	1 × H_2_	0.224 ± 0.014 *	0.181 ± 0.006	0.157 ± 0.007	0.177 ± 0.015
	CTR	0.183 ± 0.012	0.167 ± 0.026	0.155 ± 0.010	0.186 ± 0.013
TC (relative unit)	5 × H_2_	0.068 ± 0.017	0.117 ± 0.014	0.079 ± 0.010	0.081 ± 0.012
	1 × H_2_	0.068 ± 0.017	0.117 ± 0.014	0.079 ± 0.010	0.081 ± 0.012
	CTR	0.069 ± 0.011	0.090 ± 0.015	0.065 ± 0.010	0.071 ± 0.006
SB (relative unit)	5 × H_2_	5.545 ± 0.920	2.382 ± 0.246 *	2.571 ± 0.747 *	2.137 ± 0.644 *
	1 × H_2_	6.468 ± 1.731	4.096 ± 0.929 *	2.521 ± 0.897 *	3.745 ± 1.124 *
	CTR	5.054 ± 1.431	6.557 ± 1.746	4.037 ± 0.837	6.555 ± 0.725

Note: DC: diene conjugates, TC: triene conjugates, SB: Schiff’s bases, CHF: chronic heart failure. Research group 1: animals received multiple exposures to H_2_. Research group 2: animals received single exposure to H_2_. Data are presented as mean values ± SD. * *p* < 0.05, vs. control group (Student’s *t*-test). Indicators of healthy animals (physiological norm): concentration of SB—3.012 ± 0.654 (relative unit).

**Table 4:** Dynamics of the content of red blood count, hemoglobin and the mean corpuscular volume in the blood sample of rats with simulated CHF.

Indicator	Group	Day of the Experiment			
		1	3	7	14
RBC, (×10^12^/L)	5 × H_2_	3.41 ± 0.67 *	3.83 ± 0.55 *	4.12 ± 0.34	4.49 ± 0.63 *
	1 × H_2_	4.13 ± 0.74 *	4.33 ± 0.51	4.45 ± 0.36	5.51 ± 0.32
	CTR	5.46 ± 0.57	4.56 ± 0.13	4.410 ± 0.28	5.52 ± 0.31
Hb (g/L)	5 × H_2_	63.83 ± 9.11 *	76.63 ± 5.17 *	90.21 ± 5.77	82.85 ± 11.80 *
	1 × H_2_	85.47 ± 4.01 *	81.81 ± 8.32	92.61 ± 2.92	111.22 ± 3.56
	CTR	94.54 ± 5.18	87.08 ± 5.01	94.13 ± 7.01	111.16 ± 2.91
MCV, (fL)	5 × H_2_	57.42 ± 2.46 *	58.67 ± 4.65	58.41 ± 1.60	56.62 ± 0.51 *
	1 × H_2_	57.61 ± 1.21 *	59.43 ± 1.77	62.61 ± 3.08	62.01 ± 3.13
	CTR	63.03 ± 1.47	61.33 ± 1.33	61.67 ± 2.18	60.67 ± 2.18

Note: RBC: red blood cell count, Hb: hemoglobin, MCV: mean corpuscular volume, CHF: chronic heart failure. 5 × H_2_—animals received multiple H_2_ exposures; 1 × H_2_—animals received single H_2_ exposure; CTR—control group. Data are presented as mean values ± SD. * *p* < 0.05, vs. control group (Student’s *t*-test). Indicators of healthy animals (physiological norm): concentration of RBCs 5.85 ± 0.52 (×1012/L), Hb 101.2 ± 8.48 (g/L), MCV 53.2 ± 0.37 (fL) (relative unit).

## Data Availability

All data generated or analyzed during this study are included in this published article.
